# Comparison of pericapsular nerve group block and anterior quadratus lumborum block for hip fracture surgery: a randomized clinical trial

**DOI:** 10.1016/j.bjane.2025.844643

**Published:** 2025-05-23

**Authors:** Mustafa Aslan, Alper Kilicaslan, Funda Gök, Ahmet Fevzi Kekec, Tahsin Sami Colak

**Affiliations:** a Turhal State Hospital, Department of Anaesthesiology and Reanimation, Tokat, Turkey; b Necmettin Erbakan University, Meram Medical Faculty, Department of Anaesthesiology and Reanimation, Konya, Turkey; c Necmettin Erbakan University, Meram Medical Faculty, Department of Orthopedics and Traumatology, Konya, Turkey

**Keywords:** Anesthesia, Arthroplasty, Hip fractures, Nerve block, Postoperative pain

## Abstract

**Objective:**

This study compared the Pericapsular Nerve Group (PENG) block combined with the Lateral Femoral Cutaneous Nerve (LFCN) block to the anterior Quadratus Lumborum Block (QLB) in patients undergoing Total Hip Arthroplasty (THA).

**Methods:**

In this prospective, double-blind trial, 80 adults scheduled for THA under spinal anesthesia were randomized to receive either an anterior QLB (n = 40) with 30 mL of 0.25% bupivacaine or a combined PENG + LFCN block (n = 40) using 25 mL of 0.25% bupivacaine for PENG and 5 mL for LFCN. The primary outcome was cumulative 24 hour postoperative intravenous morphine consumption. Secondary outcomes included pain scores, quadriceps strength, patient satisfaction and side effects.

**Results:**

No significant differences were observed between the groups in morphine consumption or pain scores during the first 12 hours (p > 0.05). At 24 hours, the PENG + LFCN group demonstrated significantly lower morphine consumption (p = 0.027) and resting VAS scores (p < 0.001). Quadriceps weakness occurred in 15% (6/40) of anterior QLB patients at 6 hours (p = 0.026), whereas no weakness was observed in the PENG + LFCN group within 24 hours. Patient satisfaction and the incidence of complications were comparable between the groups.

**Conclusion:**

Both anterior QLB and PENG + LFCN blocks provide effective analgesia for up to 12 hours post-THA. However, the PENG + LFCN combination offers prolonged analgesia, reduced opioid requirements and better preservation of quadriceps strength.

## Introduction

Total Hip Arthroplasty (THA) is one of the most common orthopedic procedures in the United States, with over 400,000 surgeries performed annually. The numbers are expected to increase due to the aging population.^1^ There is growing interest in the perioperative analgesia of THA surgery to optimize early postoperative mobilization and discharge.^2^ Peripheral nerve and fascial plane blocks are critical to multimodal analgesia, reducing opioid use, side effects (e.g., respiratory depression, nausea), and hospital stays while accelerating mobilization.^3^^,^^4^ However, the hip joint’s complex innervation and the need to preserve motor function complicate optimal analgesia for THA and the optimal postoperative regional analgesia technique for THA remains debated.^5^ Among emerging options, two recently described motor-sparing techniques have gained prominence: the anterior Quadratus Lumborum Block (QLB)^6^ and the Pericapsular Nerve Group (PENG) block.^7^

The anterior QLB, which involves the injection of local anesthetic in the plane between the Quadratus Lumborum (QL) and Psoas Major (PM) muscles, with potential spread to the lumbar plexus, has been shown to effectively control THA pain.^8^, ^9^, ^10^ The PENG block selectively blocks sensory innervation to the anterior hip capsule ‒ a region predominantly comprised of nociceptive fibers ‒ via branches of the obturator, accessory obturator, and femoral nerves.^7^^,^^11^ Postoperative PENG blocks have been shown to reduce pain scores, opioid consumption, and the time to first mobilization following THA.^12^^,^^13^ When the PENG block is combined with the Lateral Femoral Cutaneous Nerve (LFCN) block, which provides sensory innervation to the lateral thigh, the missing dermatome blockade area is completed.^14^^,^^15^

Although a few previous studies have compared these two blocks, our study has key differences. Most are focused on elective total hip arthroplasty rather than traumatic hip fractures.^16^, ^17^, ^18^ Additionally, some did not combine the LFCN block with the PENG block,^16^^,^^17^^,^^19^^,^^20^ a combination we routinely use and recommended.^14^^,^^15^ Furthermore, one study used the lateral QLB instead of the anterior QLB,^18^ and another had a retrospective design.^20^

This study aims to compare the effectiveness of the PENG + LFCN block with the anterior QLB in reducing postoperative opioid consumption, improving analgesia, and preserving quadriceps muscle strength in patients undergoing total hip arthroplasty.

## Materials and methods

This trial was registered on ClinicalTrials.gov (NCT05654519) prior to patient enrollment. Following approval from the Institutional Review Board (IRB n° 2021/541), written and verbal informed consent was obtained from all participants. This single-center, prospective, randomized study was conducted in the operating rooms of a university-affiliated hospital. The manuscript adheres to the Consolidated Standards of Reporting Trials (CONSORT) guidelines.

Patients aged 45–85 years, with American Society of Anesthesiologists (ASA) physical status I–III, scheduled for unilateral total hip arthroplasty due to hip fracture, were included. Exclusion criteria comprised: contraindications to regional anesthesia or peripheral nerve blockade, cognitive impairment/communication barriers, weight < 50 kg or > 100 kg, Body Mass Index (BMI) > 40 kg.m^−2^ (due to concerns regarding altered anesthetic pharmacokinetics and technical challenges in block administration), peripheral neuropathy, coagulation disorders, chronic pain, severe hepatic/cardiac/renal failure, active opioid use, revision arthroplasty, diabetes mellitus, or pregnancy.

Enrollment occurred between April 2022 and April 2023. Consenting subjects were randomized to receive either the anterior QLB or the combined PENG + LFCN block using a closed opaque-envelope technique. Envelopes were opened by an independent researcher prior to block administration. All research staff, care team members (except the regional anesthesia team), and patients remained blinded to group allocation. Standardized protocols for block performance and postoperative care were implemented to minimize bias.

Preoperative assessment included the evaluation of pain intensity using the 10-point Visual Analog Scale (VAS, 0 cm = no pain, 10 cm = worst possible pain) and the explanation of how to use the Patient-Controlled Analgesia (PCA) device. Demographic data, including sex, age, height, weight, BMI, and ASA scores, were recorded for all patients. The anesthesia method and monitoring techniques used were standard routine practices with no study-specific interventions. Prior to the procedure, standard non-invasive monitoring (ECG, NIBP, and SpO_2_) was applied, and oxygen was administered via a nasal cannula while intravenous sedation was administered with 0.03 mg.kg^−1^ midazolam and 1 mcg.kg^−1^ fentanyl. To maintain blinding, aseptic skin preparation was applied to both block sites, irrespective of group assignment. A 10 cm, 21-gauge echogenic needle was used for both block groups.

### PENG block

**Figure 1:**
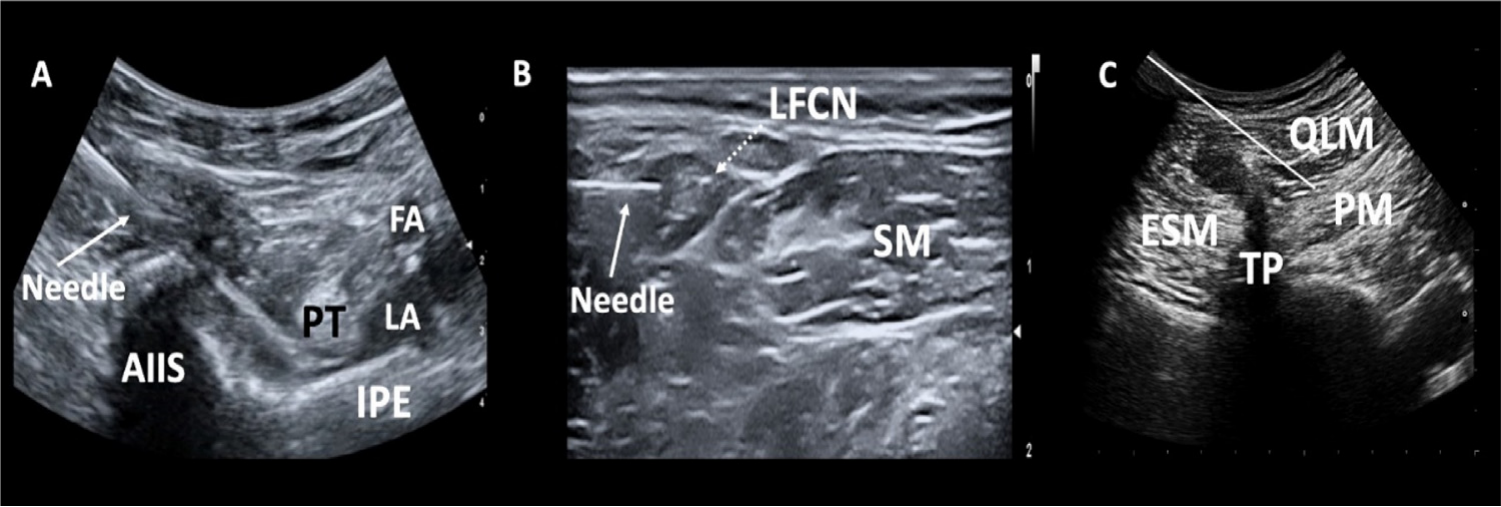
Sonoanatomy of (A) pericapsular nerve group block (B) lateral femoral cutaneous nerve block and (C) anterior quadratus lumborum block. Solid white line indicates the trajectory of the needle for local anesthetic placement. AIIS, Anterior Inferior Iliac Spine; FA, Femoral Artery; IPE, Iliopubic Eminence; PT, Tendon of Psoas muscle; LA, Local Anesthetic; LCFN, Lateral Femoral Cutaneous Nerve; SM, Sartorius Muscle, QLM, Quadratus Lumborum Muscle; ESM, Erector Spinae Muscle, PM, Psoas Muscle, TP, Transverse Process.

While the patient was in a supine position, a low-frequency convex (2‒5 MHz) transducer was used to visualize the anterior inferior iliac spine, iliopsoas tendon, iliopubic eminence, and femoral artery. As described previously, an echogenic needle was advanced laterally to medially (in-plane) until it reached the lateral and inferior edge of the iliopsoas tendon.^7^ Bupivacaine hydrochloride (25 mL, 0.25%) was then injected in 5 mL increments with intermittent negative aspiration between the iliopsoas tendon and iliopubic eminence (Fig. 1A).

### LFCN block

The LFCN was located/identified medial and inferior to the anterior superior iliac spine and laterally or superficially to the sartorius muscle. An echogenic needle was then advanced laterally to medially (in-plane) into the plane containing the nerve, and bupivacaine hydrochloride (5 mL, 0.25%) was injected. The spread of the local anesthetic around the nerve was visualized (Fig. 1B).

### Anterior QLB

This block was performed with the patient in the lateral decubitus position, with the surgical site positioned upward. A convex transducer (2‒5 MHz) was placed transversely along the mid-axillary line at the L4 level to obtain a “shamrock sign”. In this position, the QL, psoas major, and erector spinae muscles, as well as the L3 and L4 transverse processes, were visualized (Fig. 1C). The echogenic needle was advanced in-plane from posterior to anterior until it pierced the ventral fascia of the QL muscle. Bupivacaine (30 mL, 0.25%) was then injected into the plane between the QL and PM muscles, and the spread was visualized.

In cases of ineffective or incomplete blocks ‒ defined as a VAS reduction < 3 or VAS ≥ 5 at rest 30 minutes post-block ‒ it was preemptively planned to administer rescue analgesics and exclude the patient from the final analysis.

### Anesthesia and postoperative analgesia

Thirty minutes after the block procedures, the same spinal anesthetic regimen, consisting of 2.5 mL of 0.5% hyperbaric bupivacaine (12.5 mg) and 25 mcg fentanyl, was administered to all patients. In case of failure of spinal anesthesia (inadequate/absent sensory block requiring supplemental analgesia/sedation) general anesthesia was applied, and the patient was excluded from the study.

Standardized postoperative care orders were implemented in the PACU as part of a multimodal analgesia protocol: all patients received 0.1 mg.kg^−1^ Intravenous (IV) dexamethasone (maximum 8 mg) and 1000 mg IV paracetamol. For the first 24 hours postoperatively, 10 mg.kg^−1^ IV paracetamol (maximum 1000 mg) was administered every 6 hours, supplemented with morphine via Patient-Controlled Analgesia (PCA) (1 mg bolus with a 10-minute lockout interval) as rescue medication.

At the 24^th^ postoperative hour, PCA was discontinued, and oral paracetamol was continued until discharge, within the multimodal analgesia protocol. For breakthrough pain (VAS > 3), tramadol 1 mg.kg^−1^ (administered at ≥ 4-hour intervals, maximum 300 mg.day^−1^) was used as the first-line rescue analgesic.

### Primary and secondary outcome measures

• Primary Outcome:

Cumulative morphine consumption within the first 24 hours postoperatively, measured at predefined intervals (4, 12, and 24 hours).

• Secondary Outcomes:

1. Pain Intensity:
  - At rest: Assessed preoperatively (baseline), 30 minutes post-block, and at 4, 12, and 24 hours postoperatively using the Visual Analog Scale (VAS).
  - Movement-evoked pain: Evaluated at 24 hours postoperatively using a standardized walk test, per institutional surgical protocol (mobilization delayed until 24 hours).
2. Quadriceps muscle strength was measured via isometric knee extension at 6, 12, and 24 hours postoperatively. This assessment was conducted in a standardized supine position (hips flexed at 45°, knees at 90° flexion) without requiring active mobilization (e.g., standing/walking).^21^
3. Patient satisfaction was rated at 24 hours postoperatively using a 5-point Likert scale: 1 = Terrible, 2 = Poor, 3 = Satisfactory, 4 = Good, 5 = Excellent.
4. Adverse effects were documented between 0 and 24 hours postoperatively, including nausea, vomiting, pruritus, respiratory depression (respiratory rate ≤ 8 min), and urinary retention. No interim analysis was performed.

### Statistical analysis

No previous studies have compared pain scores between patients receiving the PENG block and anterior QLB. Sample size calculation for our study was based on a study by He et al.,^22^ which compared cumulative opioid consumption in patients who received anterior QLB after THA. The cumulative morphine consumption in the anterior QLB group was 16 mg over 24 hours. With a 5% alpha error and 80% power, a 15% reduction in cumulative opioid consumption was expected after the PENG block. The minimum required sample size per group was 36 patients. Considering potential dropouts and variability in standard deviation, we calculated a sample size of 40 patients per group.

Data were presented as percentages (%), frequencies (n), mean ± Standard Deviation (SD), minimum, median, and maximum values, with no missing data. The Chi-square and Fisher's exact tests were used for categorical variables, while independent *t*-tests were used for normally distributed parametric data. For non-parametric data, the Mann-Whitney *U* test was applied. All statistical analyses were performed using SPSS for Windows version 22. A significance level of p < 0.05 was considered statistically significant.

## Results

**Figure 2:**
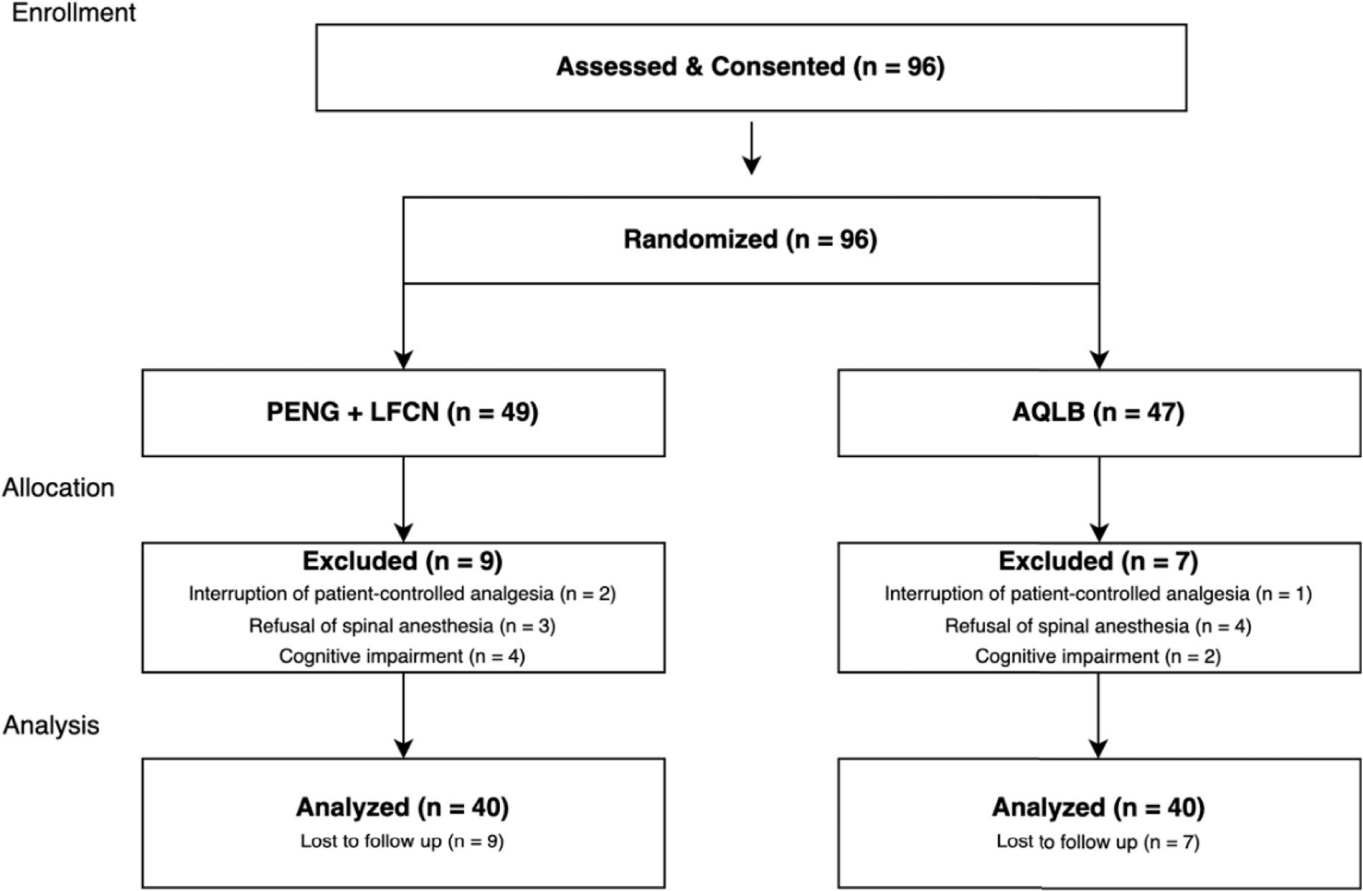
Consolidated Standards of Reporting Trials flow diagram. LFCN, Lateral Femoral Cutaneous Nerve; PENG, Pericapsuler Nerve Group; AQLB, Anterior Quadratus Lumborum Block.

During the study period, data from 96 patients were recorded. Some patients were excluded due to the presence of cognitive impairment, refusal of spinal anesthesia, or technical issues with the PCA device (Fig. 2). The final study population consisted of 80 subjects, with equal numbers in each group.

Participant demographic and baseline characteristics (sex, age, BMI, ASA score, surgical duration, and approach) are summarized in Table 1, and there were no significant differences between the groups (p > 0.05).

**Table 1:** Baseline characteristics of patients.

	AQLB (n = 40)	PENG (n = 40)	p-value
**Sex (male), n (%)**	17 (42.5)	19 (47.5)	0.653^a^
**Age (years), mean ± SD**	69.55 ± 6.06	68.95 ± 8.09	0.708^b^
**BMI (kg.m^−2^), mean ± SD**	28.67 ± 3.68	27.65 ± 3.24	0.190^b^
**ASA (1/2/3), n (%)**	0/19 (47.5) /21 (52.5)	2 (5) /15 (37.5) /23 (57.5)	0.299^c^
**Duration of Surgery, mean ± SD**	155.53 ± 18.77	150.07 ± 19.19	0.203^b^
**Surgical approach**			
Right / Left, n (%)	24 (60) /16 (40)	20 (50)/20 (50)	0.369^a^

ASA, American Society of Anesthesiologists; PENG, Pericapsular Nerve Group + Lateral femoral cutaneous nerve block, AQLB, Anterior Quadratus Lumborum Block; BMI, Body Mass Index.

a Chi-Square analysis.

b *t*-test.

c Fisher’s Exact test.

### Primary outcome

**Figure 3:**
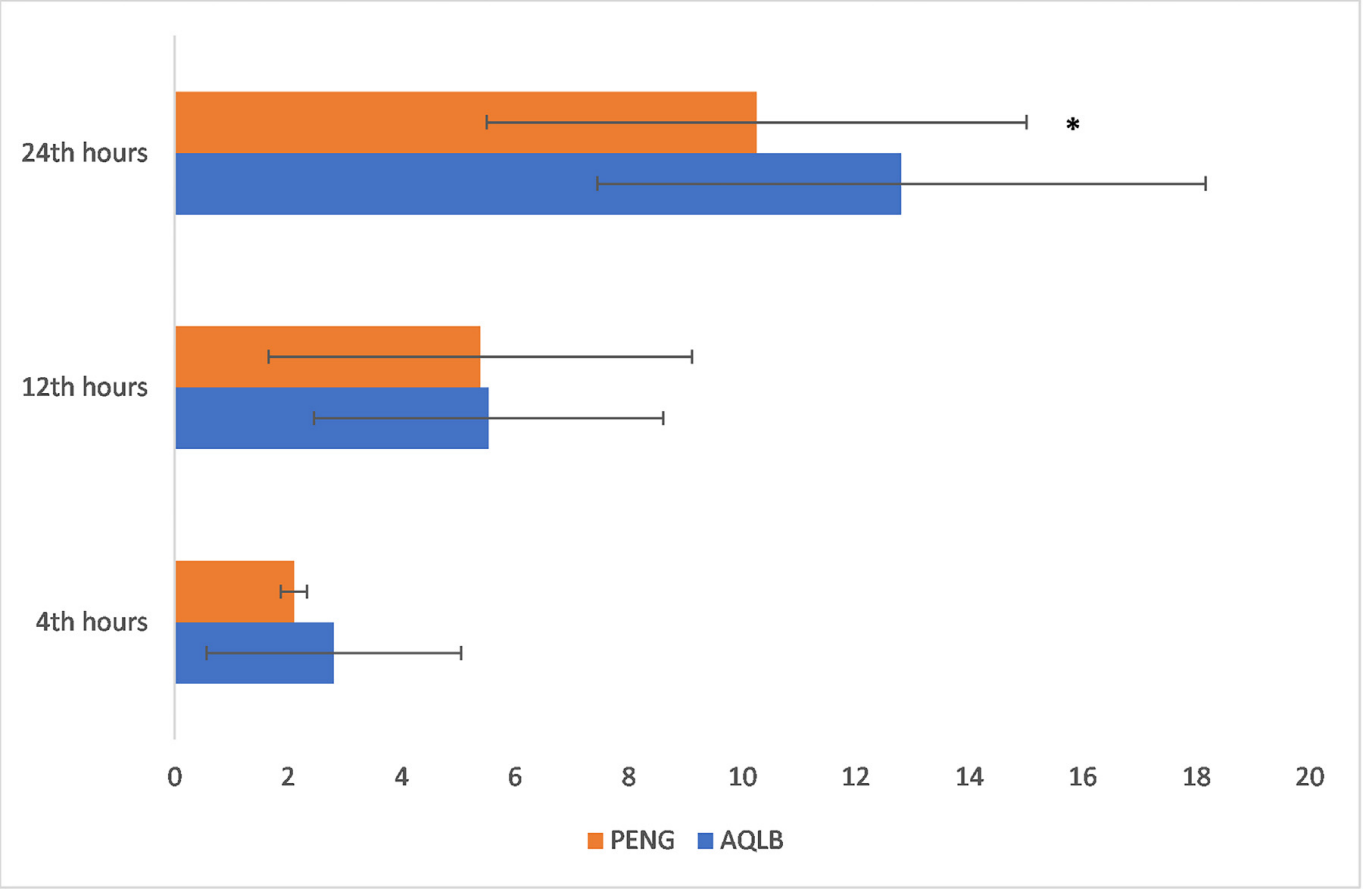
Comparison of the cumulative morphine consumption among the study groups. PENG, Pericapsuler Nerve Group + Lateral Femoral Cuteneous Nerve Block; AQLB, Anterior Quadratus Lumborum Block. *p = 0.027.

The cumulative opioid consumption over 24 hours postoperatively is shown in Figure 3. No significant differences were found between the groups at 4 and 12 hours postoperatively. However, at the 24-hour postoperative mark, the cumulative intravenous morphine consumption in the PENG + LFCN group was significantly lower than in the anterior QLB group (10.25 ± 4.76 vs. 12.80 ± 5.36, Cohen’s *d* = 0.50, 95% Confidence Intervals 0.1 to 0.9; p = 0.027) (Fig. 3).

### Secondary outcomes

**Figure 4:**
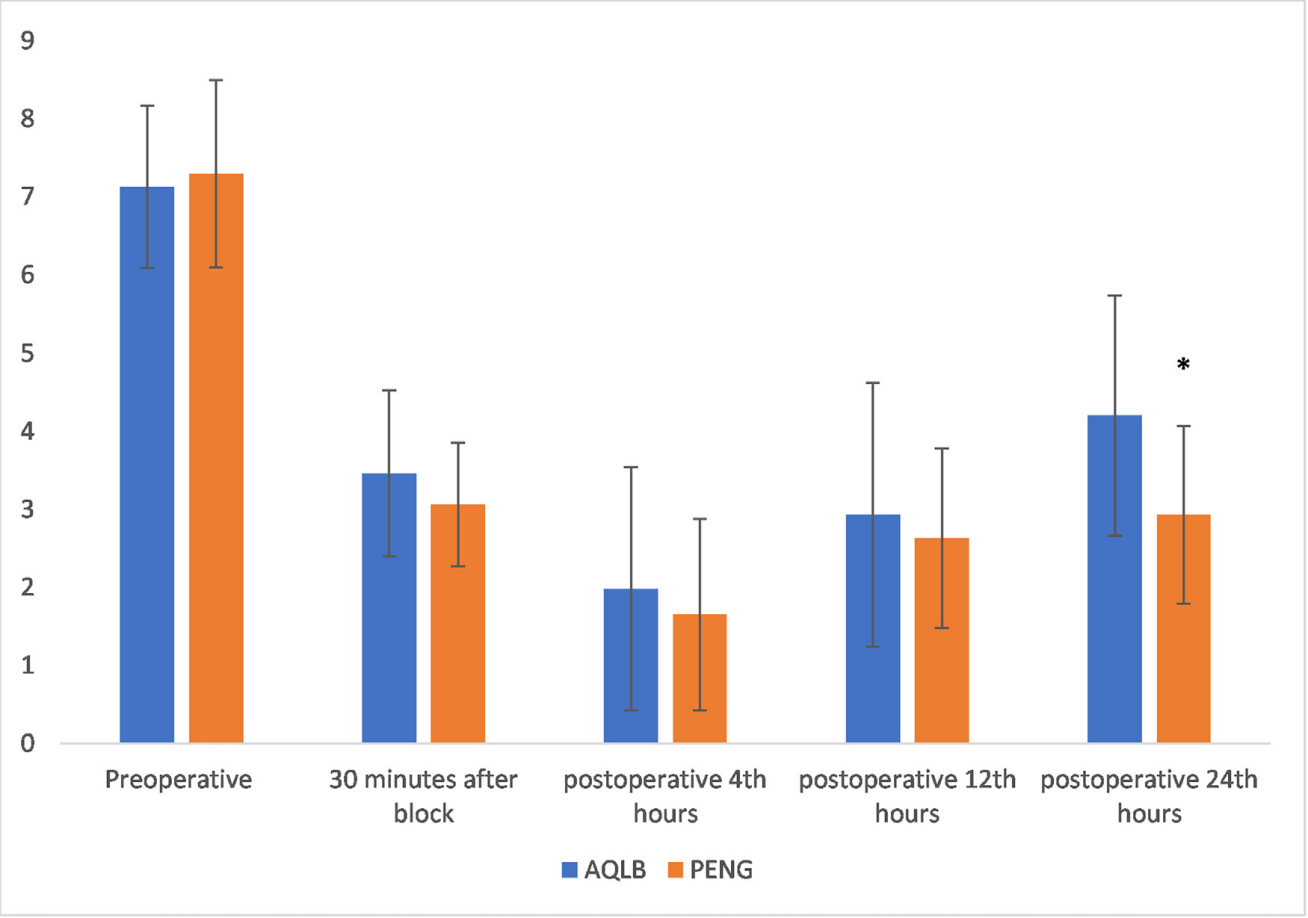
Comparison of the pain scores among the study groups. PENG, Pericapsuler Nerve Group + Lateral Femoral Cuteneous Nerve Block; AQLB, Anterior Quadratus Lumborum Block. * p < 0.05.

Visual Analog Scores (VAS) at 24 hours postoperatively, the resting VAS scores in the PENG + LFCN group were significantly lower compared to the anterior QLB group (2.93 ± 1.14 vs. 4.20 ± 1.54, Cohen’s *d* = 0.94, 95% Confidence Intervals 0.47 to 1.40; p < 0.001) (Fig. 4). Other VAS scores measured at rest and during movement at different time points were similar between the two groups (p > 0.05). Quadriceps weakness occurred in 15% (6/40) of anterior QLB patients at 6 hours, whereas no weakness was observed in the PENG + LFCN group within 24 hours (Odds Ratio = 0.06, 95% CI 0.3 to 1.1; p = 0.026). No quadriceps weakness was observed at any other time points in either group.

There were no significant differences between the groups in terms of opioid-related side effects, including nausea (p = 0.59), vomiting (p = 0.74), or pruritus (p = 0.50). No adverse events or complications were observed in either group. Furthermore, patient satisfaction scores were comparable between the groups (Table 2).

**Table 2:** Additional outcomes of interest.

Postoperative		AQLB (n = 40)		PENG (n = 40)		p-value
		n	%	n	%	
Quadriceps Weakness	6^th^ Hours	6	15	0	0.0	0.026^a^
	12^th^ Hours	0	0.0	0	0.0	
	24^th^ Hours	0	0.0	0	0.0	
Patient Satisfaction	Unsatisfied	0	0.0	0	0.0	0.280^a^
	Satisfied	5	12.5	4	10.0	
	Good	22	55.0	16	40.0	
	Excellent	13	32.5	20	50.0	
Nousea		10	25	8	20	0.59^a^
Vomiting		5	12.5	6	15	0.74^a^
Pruritus		3	7.5	2	5	0.50^a^

AQLB, Anterior Quadratus Lumborum Block; PENG, Pericapsular Nerve Group + Lateral Femoral Cutaneous Nerve Block.

a Chi-Square analysis.

## Discussion

This study compared the postoperative pain scores, morphine consumption, and quadriceps muscle strength between PENG and LFCN blocks versus anterior QLB in patients undergoing THA after hip fracture. Patients in the PENG + LFCN group had lower resting VAS scores and consumed less morphine at 24 hours postoperatively compared to the anterior QLB group. Additionally, quadriceps weakness was detected in 15% of the anterior QLB group during early postoperative hours.

In a study by He et al., which included 88 patients undergoing hip arthroplasty, the analgesic efficacy and safety of anterior QLB were compared to a control group. They found that postoperative resting and dynamic VAS scores were significantly lower in the anterior QLB group until 48 hours postoperatively.^22^ Nassar et al. compared the analgesic effectiveness and motor block profiles of transmuscular QLB and Suprainguinal Fascia Iliaca Block (SIFIB) in hip arthroplasty patients and found that both groups had similar postoperative pain scores and analgesia durations, with lower opioid consumption in the SIFIB group.^23^

Chung et al. demonstrated that PENG block significantly reduced cumulative opioid consumption and pain scores at 24 hours after hip surgery.^24^ Mosaffa et al. compared the postoperative analgesic effectiveness of PENG block and FIKB in hip fracture surgery. They reported that 15 minutes post-block and at 12 hours postoperatively, the PENG block group had lower VAS scores and less opioid consumption over the 24-hour postoperative period.^25^ Huda et al. conducted a meta-analysis and found that PENG block significantly reduced 24-hour opioid consumption after hip surgery, delayed the time to the first analgesic request, and resulted in less motor block risk.^26^ Aliste et al. compared PENG block to SFIB in 40 patients undergoing THA under spinal anesthesia and found that the PENG block group had lower quadriceps motor block at 3 hours (45% vs. 90%) and 6 hours (25% vs. 85%).^27^

Previous studies comparing anterior QLB and PENG blocks in hip surgery have shown similar outcomes, although some contradictory results have been reported. Differences in drugs, volumes, anesthesia methods, and whether LFCN block was included or not may have contributed to these results, as there is no standardization in the methodology. In a study by Tayfun Et et al., which compared PENG, anterior QLB, and intra-articular blocks for primary THA, they found similar analgesic effects between PENG and anterior QLB.^16^ This may be due to differences in drug volumes (30 mL of 0.5% bupivacaine for the anterior QLB group vs. 20 mL of 0.5% bupivacaine for the PENG block group) and the exclusion of LFCN block in the PENG group. Similar to our study, they reported better preservation of quadriceps muscle strength postoperatively in the PENG group compared to anterior QLB.

Braun et al. performed a retrospective study comparing PENG and anterior QLB after THA and found no difference in morphine consumption at 24 and 48 hours postoperatively.^20^ Abdelsalam et al. compared PENG and anterior QLB methods in hip arthroplasty and found no differences in resting and dynamic pain scores, cumulative opioid consumption, or time to first analgesic request between the two groups.^17^ In these studies, unlike ours, LFCN block was not added to the PENG block.

Wang et al. reported significantly lower maximum pain scores in the PENG group and significantly lower pain scores at 3 hours after surgery at rest and during movement at 3 and 6 hours postoperatively. However, they found no significant differences in morphine consumption, hospital length of stay, pain levels one year postoperatively, or complication incidence between the groups.^19^ Both groups did not show quadriceps weakness. Hay et al. compared PENG and lateral QLB after THA and observed lower cumulative opioid consumption and lower pain scores during movement between 36 and 72 hours postoperatively in the lateral QLB group.^18^

Ritesh Roy et al. concluded that combining PENG block with LFCN block provided superior analgesia with lower pain scores than PENG block alone.^15^ In our study, we found that the addition of LFCN block to the PENG block resulted in prolonged analgesic duration and reduced morphine consumption. We hypothesize that without the LFCN block, the PENG block alone may provide incomplete dermatomal blockade, resulting in insufficient analgesia.

In this study, we observed lower quadriceps strength at 6 hours postoperatively in the anterior QLB group when compared to the PENG + LFCN group. This is likely due to the fact that the PENG block only targets the joint branches of the FN, ON, and AON. On the other hand, higher volumes or intramuscular needle placement during PENG block might result in unintended spread and quadriceps weakness.^28^ One possible explanation for these results is that the better vascularization of the anterior QLB region may lead to a shorter duration of analgesia. Additionally, increased drug diffusion toward neural structures could contribute to motor blockade. At the L4 vertebral level, when a local anesthetic is injected between the Quadratus Lumborum (QL) and Psoas Major (PM) muscles, it may spread medially toward the ventral rami of L2 and L3, laterally toward the lateral cutaneous nerve of the thigh, and caudally beneath the fascia iliaca.^29^ However, previous studies have reported an inconsistent distribution following anterior QLB, which may explain both the variable outcomes observed in prior research and the motor weakness seen in some patients in this study.^29^^,^^30^

### Limitations

This study had some limitations. First, the effect of spinal anesthesia may have influenced the early postoperative assessment of motor strength. Second, the study did not include a normal control group. However, both blocks have been previously compared with control groups, showing superior results compared to placebo. Third, although the study was prospective and randomized, and preoperative sedation was administered, patients may not have been completely blinded since they were awake during the block procedure. However, based on postoperative assessment questions, we found that patients were unaware of which block was performed. Fourth, information on pain scores during movement before the 24-hour mark, discharge times, pain scores and analgesic consumption after 24 hours could not be obtained.

## Conclusions

In conclusion, while both anterior QLB and PENG + LFCN blocks are effective analgesic methods for up to 12 hours postoperatively in patients undergoing THA after fracture, our findings suggest that the PENG + LFCN combination provides significantly longer-lasting analgesia, preserves quadriceps muscle strength, and reduces opioid consumption compared to anterior QLB. Based on these results, the PENG + LFCN block may be a preferable option for THA analgesia, particularly in clinical settings prioritizing early mobilization and opioid-sparing strategies. However, further multicenter studies with larger sample sizes are needed to confirm these findings and determine the clinical significance of the differences.

## Authors contributions

All authors contributed to the study conception and design. Material preparation, data collection and analysis were performed by Mustafa Aslan, Alper Kilicaslan, Funda Gok, Ahmet Fevzi Kekec, Tahsin Sami Colak. The first draft of the manuscript was written by Mustafa Aslan and all authors commented on previous versions of the manuscript. All authors read and approved the final manuscript.

## Conflicts of interest

The authors declare no conflicts of interest.
